# Transcriptional Response of Two Core Photosystem Genes in *Symbiodinium* spp. Exposed to Thermal Stress

**DOI:** 10.1371/journal.pone.0050439

**Published:** 2012-12-07

**Authors:** Michael P. McGinley, Matthew D. Aschaffenburg, Daniel T. Pettay, Robin T. Smith, Todd C. LaJeunesse, Mark E. Warner

**Affiliations:** 1 College of Earth, Ocean, and Environment, University of Delaware, Lewes, Delaware, United States of America; 2 Department of Biology, Florida International University, Miami, Florida, United States of America; 3 Department of Biology, The Pennsylvania State University, University Park, Pennsylvania, United States of America; King Abdullah University of Science and Technology, Saudi Arabia

## Abstract

Mutualistic symbioses between scleractinian corals and endosymbiotic dinoflagellates (*Symbiodinium* spp.) are the foundation of coral reef ecosystems. For many coral-algal symbioses, prolonged episodes of thermal stress damage the symbiont's photosynthetic capability, resulting in its expulsion from the host. Despite the link between photosynthetic competency and symbiont expulsion, little is known about the effect of thermal stress on the expression of photosystem genes in *Symbiodinium*. This study used real-time PCR to monitor the transcript abundance of two important photosynthetic reaction center genes, *psbA* (encoding the D1 protein of photosystem II) and *psaA* (encoding the P_700_ protein of photosystem I), in four cultured isolates (representing ITS2-types A13, A20, B1, and F2) and two *in hospite Symbiodinium* spp. within the coral *Pocillopora* spp. (ITS2-types C1b-c and D1). Both cultured and *in hospite Symbiodinium* samples were exposed to elevated temperatures (32°C) over a 7-day period and examined for changes in photochemistry and transcript abundance. *Symbiodinium* A13 and C1b-c (both thermally sensitive) demonstrated significant declines in both *psbA* and *psaA* during the thermal stress treatment, whereas the transcript levels of the other *Symbiodinium* types remained stable. The downregulation of both core photosystem genes could be the result of several different physiological mechanisms, but may ultimately limit repair rates of photosynthetic proteins, rendering some *Symbiodinium* spp. especially susceptible to thermal stress.

## Introduction

The widespread and long-term persistence of coral reef ecosystems dominated by calcifying cnidarians is attributed to the mutualistic symbiosis between scleractinian corals and endosymbiotic members of the dinoflagellate genus, *Symbiodinium* [1]. These dinoflagellates provide the host with a source of photosynthetically derived simple carbon compounds (e.g. glycerol) that account for a significant proportion of the energy required for their daily metabolic demands [2]. Coral reef ecosystems have been an enduring feature of warm tropical waters for millions of years, but in recent decades, a dramatic decline in coral coverage and diversity has threatened their existence worldwide due to an increasing occurrence of coral bleaching events [3], [4]. Coral bleaching is a phenomenon characterized by a significant loss in photosynthetic pigments and/or expulsion of the symbiotic algae, ultimately reducing the translocation of photosynthetically fixed carbon to the animal host [2]. Although coral bleaching can be the result of many physical and biological stressors [5], elevated sea surface temperatures due global climate change promote a breakdown in the coral-algal symbiosis and are generally considered a significant threat to the persistence of coral reefs [4], [6], [7]. The long-term impacts of bleaching include reduced rates of calcification and growth, lowered fecundity, increased proliferation of disease, and mass mortality, which eventually degrade the ecosystem's diversity and function [8]–[11].

The underlying molecular and physiological mechanisms that initiate temperature-induced coral bleaching remain an area of considerable research. Extensive evidence suggests that a collapse in the symbiosis can be attributed to an impairment of the photosynthetic machinery within the symbiont [5]. Elevated temperatures reduce the efficiency of photochemistry by imposing damage to several photosynthetic proteins, including photosystem II (PSII) [12], [13], light harvesting complexes [14], the Calvin cycle [15], [16], or the thylakoid membrane [17], [ but see 18]. Understanding the proximal site or exact driver of thermal damage is confounded by the considerable diversity within the genus *Symbiodinium*, Currently, this group is divided into nine divergent phylogenetic clades (designated A-I) [19], [20]. Furthermore, each clade consists of numerous distinct *Symbiodinium* genotypes that display functional differences in ecological and physiological attributes, including bleaching susceptibility [21], [22]. Recent evidence also suggests that there is variability within the initial location of thermal-damage between different types of *Symbiodinium* [23].

In all oxygenic photosynthetic organisms, there is a tightly-coupled balance between the rate of light-induced damage to photosynthetic proteins and the rate of subsequent cellular repair [24], [25]. Specifically, in order to avoid an accumulation of damaged PSII reaction centers, photosynthetic organisms have evolved several repair cycles that involve the proteolytic breakdown, synthesis, and re-insertion of the D1 subunit (PsbA, which is encoded by the chloroplast gene *psbA*) into the PSII protein complex [25], [26]. As the D1 is one of the key structural proteins of the PSII reaction center core and responsible for providing the plastoquinone (Q_B_) binding pocket, it has a pivotal role in maintaining the intact photosynthetic electron transport chain. Of particular importance for *Symbiodinium*, periods of elevated temperature disrupt the cellular maintenance of the D1 protein, either by inhibiting the overall rate of protein repair [12], [27] or by inducing an accelerated rate of PSII photo-inactivation [13]. This imbalance triggers a condition known as photoinhibition, in which a rapid accumulation of photodamaged D1 ultimately reduces the content of functional PSII reaction centers, thereby prompting photosynthetic dysfunction. These episodes of chronic photoinhibition are acknowledged as a driving factor responsible for many instances of large-scale coral bleaching [12], [28], [29]. While there is less evidence for photoinhibition of photosystem I (PSI) and subsequent degradation of the two core PSI reaction center core heterodimer proteins that are encoded by the chloroplast genes *psaA* and *psaB* [30], chilling stress can lead to photoinhibition and PSI protein loss in some plants [31], [32]. In contrast, a recent study has noted a significant decline in the stromal PSI protein, PsaC, in *Symbiodinium* within the reef coral *Turbinaria reniformis* exposed to 32°C. However, this decline was attributed to a possibly protective reduction in PSI content, as PSI electron flow was not hampered while PSII electron transport was [33].

Establishing how *Symbiodinium* spp. regulate their photosynthetic machinery under elevated temperatures is vital towards understanding the underlying mechanisms contributing to coral bleaching. While previous work has focused on the breakdown and repair of some photosynthetic proteins [13], [34]–[36], no studies have examined the expression of any chloroplast-encoded genes within *Symbiodinium* in response to thermal stress. Furthermore, the chloroplast of peridinin-dinoflagellates (e.g. *Symbiodinium*) is unique from all other photoautotrophs, as its genome is highly reduced and organized on individual “minicircles” (∼2–3 kbp) [37], emphasizing that the functional control of these genes in these dinoflagellates may not be analogous to other photosynthetic systems with regards to their maintenance or expression [38], [39]. Therefore, this study investigated the possibility of thermal alteration of the transcriptional response of two plastid genes, *psbA* and *psaA*, responsible for encoding core structural reaction center proteins located within photosystems II and I, respectively. The experiments monitored the photophysiology and transcript abundance of each gene using four cultured isolates and two *in hospite Symbiodinium* populations during simulated thermal stress events. This investigation provides evidence that, in some cases, the response to thermal stress is modulated at the transcriptional level, yet alternative points involving translational processes may also play a role within the unique chloroplasts of different *Symbiodinium* spp.

## Materials and Methods

### Culturing Conditions

Four *Symbiodinium* cultures from three divergent clades (A, B, and F) were grown in artificial seawater media (ASP-8A) [40] at 28°C on a 14∶10 light∶dark cycle in an environmental incubator (Percival Scientific, Inc.). Denaturing gradient gel electrophoresis (DGGE) of the ribosomal ITS2 region identified these cultures as A13 (formerly A1.1), A20, B1 and F2 (Culture IDs: 80 (A13), RD04 (A20), 13 (B1), and 133 (F2)) [41]. DGGE analysis was performed before and after each experiment to ensure that each *Symbiodinium* genotype remained consistent throughout the study. A20 is a putatively free-living *Symbiodinium* isolated from Caribbean environmental samples (LaJeunesse, personal communication; GenBank Accession: EU449053). Replicate flasks (n = 4) of each culture were gently bubbled with air passed through a carbon column to stabilize pH [42] and maintained semi-continuously in log-phase growth (fresh media replenished every 3–4 days) for at least two-weeks prior to the experiment. The environmental incubator simulated a sinusoidal light curve that gradually elevated the light intensity until mid-day, followed by a slow decline until the end of the cycle (75–165 µmol photons·m^−2^·s^−1^). These conditions attempt to replicate the natural progression of light intensity observed throughout a typical day from 08:00 to 22:00 (14∶10 light∶dark cycle; Figure S1).

### Thermal Stress: Cultured *Symbiodinium*

In addition to their genetic identity, three of the *Symbiodinium* isolates were previously categorized as either thermally tolerant (F2) or thermally sensitive (A13 and B1) based on previous evidence of either stability or disruption in PSII photochemistry as well as protein repair of the D1 protein within PSII [43]. Additionally, type A20 (originally isolated by J. Rodgers (EPA)) was chosen as another thermally tolerant isolate based on preliminary temperature stress experiments. The cultures were treated by elevating the temperature from 28°C to 32°C at a rate of 1°C per day. Once the temperature reached 32°C, replicate flasks (n = 4) were sampled for RNA analysis (see details below) twenty-four hours later and then, every other day (48 h) for one week. All samples were removed at 10:00, which represented the 85 µmol photons·m^−2^·s^−1^ phase of the light cycle. Additionally, chlorophyll *a* fluorescence was measured at each time point to assess the photophysiology of each culture in response to the thermal stress. Following this experiment, new flasks of untreated *Symbiodinium* A13 were exposed to the same experimental conditions (i.e. 28°C to 32°C at a rate of 1°C per day); however, samples for RNA transcript analysis were only removed every 24 h (at 10:00 each day) during the initial rise in temperature to investigate the possibility of initial changes in transcript abundance during the thermal ramping. All gene expression profiles are shown relative to a set of untreated controls that were held at 28°C.

### Thermal Stress: *In Hospite Symbiodinium*

Coral fragments from different colonies of *Pocillopora* (*verrucosa* morphospecies; [44]) were collected from Punta Galeras Reef, La Paz, MX (24°21′15′N, 110°17′05″W) and transported back to the laboratory at the Universidad Autónoma de Baja California Sur in July 2007. Colony selection was based on the symbiont composition of each coral, which was previously characterized by ITS-2 rDNA fingerprinting in 2006 [45], resulting in a total of 8 distinct colonies harboring either *Symbiodinium* C1b-c or D1 (n = 4 colonies for each symbiont). Further identification of the dominant *Symbiodinium* type from each colony was analyzed (DGGE-ITS2) again immediately before and after the experiment and no change in the specific symbiotic combinations were noted (data not shown). Six fragments from each *Pocillopora* colony were mounted to labeled 1.5-inch PVC pipe couplers with marine epoxy and divided among replicated control and treatment tanks (i.e. three fragments per colony in each treatment). Each tank was continually filled with natural seawater pumped from a shallow bay nearby and mechanically filtered through a sand bed. After a short 36 h acclimation period, the two experimental tanks were heated approximately by 1.5°C per day for 4 days and maintained at 32°C for 7 days. Conversely, the control tanks remained at the ambient seawater temperature (∼26°C) for the duration of the heating treatment. During the experiment, all coral fragments were exposed to similar midday light levels as those measured at the reef site (950–1000 µmol photons·m^−2^·s^−1^) by applying an overhead shade cloth. Total RNA was extracted (see details below) from four fragments in each treatment on days 1, 6, and 7 (final sampling point) at 32°C. To account for any differences in the diel expression of *Symbiodinium* genes, all samples were isolated/processed beginning at 09:00 each sampling day. All gene expression profiles are shown relative to the unheated controls (ambient tank), sampled at the same time and day as the thermally treated corals for each *Symbiodinium* type.

### RNA Extractions and cDNA Synthesis

Total RNA was isolated from all *Symbiodinium* samples with a commercial extraction kit (TRI Reagent Solution, Ambion) that included the optional steps in the manufacturer's protocol to remove additional contaminating proteoglycans and polysaccharides. All cultured *Symbiodinium* samples were filtered onto 5.0 µm polycarbonate filters (15–30 mL) during their exponential growth phase, and then immediately submerged in 1 mL of TRI Reagent and stored at −80°C. *Pocillopora* tissue was removed from the skeletons using a WaterPik® with ∼50 mL of 0.45 µm filtered seawater. The resulting homogenate was centrifuge at 5000 g for 5 min to isolate the *Symbiodinium* pellet. The supernatant was discarded and the pellet was resuspended and washed twice in 5 mL of filtered seawater. Each *Symbiodinium* pellet was frozen in liquid nitrogen and stored at −80°C until the RNA extractions. In order to fully lyse the *Symbiodinium* cells, samples were homogenized in the TRI Reagent with 1.0 mm glass beads for 2 minutes at full speed in a Mini Beadbeater (BioSpec Products). The resulting RNA pellets were resuspended in 50 µL nuclease-free H_2_O and quantified on a NanoDrop 2000 (Thermo Scientific). RNA samples (∼500 ng total RNA) were treated with RQ1 RNase-Free DNase (Promega) according to the manufacturer's instructions to remove any residual DNA and reverse transcription reactions were performed with a High-Capacity cDNA Reverse Transcription kit (Applied Biosystems) using random primers to initiate cDNA synthesis. Additionally, reactions without the Reverse Transcriptase enzymes were carried out for each sample and subjected to PCR to ensure the removal of all residual DNA from each RNA extraction. None of these samples were successfully amplified, thus, confirming the efficacy of the DNase treatment. The resulting cDNA was diluted 1∶5 with nuclease-free H_2_O and used as the template in the real-time PCR analysis.

### Primer Design

Table 1 presents a list of the real-time PCR primers used in this study. The Proliferating Cell Nuclear Antigen (PCNA) primers were previously established for *Symbiodinium* [46], while S-adenosyl-L-methionine synthetase (SAM) primers were selected due to their high expression stability in other *Symbiodinium* types exposed to both temperature and light stress [47]. Primers for *psbA* and β-Actin were designed based on sequence alignments of cDNA from multiple *Symbiodinium* types, as well as, several other dinoflagellates obtained from the NCBI GenBank database (www.ncbi.nlm.nih.gov). Multiple sequence alignments were performed in BioEdit [48] using the ClustalW Multiple Alignment tool [49]. Primer sets were designed from these alignments within conserved regions identified for each gene using DNASTAR (Lasergene Primer Select). Primer compatibility was tested against DNA isolated from distinct *Symbiodinium* cultures that span five different clades (ITS2-types: A13, A20, B1, C1b-c, D1 and F2). An additional *psbA* primer set was designed for the *in hospite Symbiodinium* analysis due to the amplification of a second product (also derived from the *psbA* gene; data not shown) in C1b-c samples. Furthermore, the β-Actin primers failed to amplify within *Symbiodinium* A20, while the PCNA primers only weakly amplified cDNA from the D1 alga.

**Table 1:** Target gene, encoded protein, and primer sequence used for real-time PCR.

Target Gene	Protein	Primer Sequence (5′-3′)
*psbA*	D1 protein in photosystem II	Culture *Symbiodinium*:F: GAGTAGCTGGAGTATTTGGTGGATR: TGAAGGCTACGAGAGTTATTGAAGIn Hospite *Symbiodinium*:F: TGCAGAAACTGCAGGAGATATTAGCCR: TACTCCAAGGGCAGTGAACC
*psaA*	P_700_ protein in photosystem I	F: AAGAATTGGGAACAGCAGATR: TCACAGGGATAAGTAAAACCAA
Actin	ß-Actin	F: GAGATGAAGGCTGCATCTGAGAGR: ACAAAGCTTGGCTGGAACA
PCNA	Proliferating Cell Nuclear Antigen	F: GAGTTTCAGAAGATTTGCCGAGATR: ACATTGCCACTGCCGAGGTC
SAM	S-adenosyl-L-methionine Synthetase	F: GCCTACATTTGCCGACAGATGR: AATGGCTTGGCAACACCAAT

F designates the forward primer; R designates the reverse primer.

Given the absence of any *psaA* sequence data for *Symbiodinium*, the *psaA* amino-acid sequence from the following microalgae were obtained from GenBank to create a multiple-sequence protein alignment: *Glaucocystis nostochinearum* (AAX82908.1), *Gonyaulax polyedra* (ABB89032.1), *Heterocapsa triquetra* (AAD44698.1), *Heterocapsa niei* (AAX82907.1), *Amphidinium operculatum* (CAB75844.1), *Gymnodinium mikimotio* (BAA86297.1), *Neoceratium horridum* (CAF32304.1), *and Akashiwo sanguinea* (AAX82906.1). Degenerate primers were designed (Forward (5′-3): CCTTCGGCCTGTACATCCAYAAYGAYAC; Reverse (5′3′): TGCAGTGGATTGTGAAGGCRTGNAYRTG) from the resulting protein alignment with CODEHOP (consensus-degenerate hybrid oligonucleotide primers; http://bioinformatics.weizmann.ac.il/blocks/codehop.html), and used to successfully amplify a 366 bps product from a *Symbiodinium* B1 (Conditions: 92°C for 3 min; 35 cycles of 92°C for 45 s, 61°C for 45 s, and 72°C for 45 s). The PCR product was cloned with a TOPO® TA Cloning kit with One Shot® Chemically Competent cells (Invitrogen) and bi-directionally sequenced using a BigDye® Terminator v1.1 Cycle Sequencing kit (Applied Biosystems). A BLAST search (blastn) of GenBank returned over 50 known *psaA* sequences with E-values less than 10^−15^. These hits matched sequences from various photosynthetic organisms, including the species that constructed the original protein alignment. Real-time PCR primers were developed from the resulting *psaA* sequence using DNASTAR Lasergene Primer Select and successfully amplified across several *Symbiodinium* samples spanning different clades (A, B, C, D and F).

### Real-Time PCR and Gene Expression Analysis

Real-time PCR assays were performed on an ABI Prism 7500 Sequence Detection System (Applied Biosystems). Three technical replicates of each cDNA sample were amplified using 2 µL H_2_O, 5 µL SensiMix SYBR mastermix (Bioline), 1 µL of each forward and reverse primer (optimized final concentrations of 0.9 µM and 0.3 µM, respectively, for all primers), and 1 µL cDNA in 10 µL total volume reactions. PCR conditions consisted of 50°C for 2 min, 95°C for 10 min, followed by 40 cycles of 95°C for 15 s, 53°C for 30 s (48°C for the *psaA* primers), and 72°C for 1 min. Following each reaction, a dissociation step (melt curve analysis) from 60°C to 95°C was included to ensure that primer dimers did not significantly contribute to the fluorescence signal. In addition to the experimental samples, a five-step ten-fold dilution series of cDNA from a common control sample was included on each plate for every primer set to account for differences in priming efficiency between plates. A fixed fluorescent threshold of 0.05 was applied for all reactions to determine the cycle threshold (C_T_) values. The resulting C_T_ values of the technical replicates were averaged and used only if their standard deviation was less than 0.50 (typically less than 0.25). Additionally, the expression stability of the three reference genes under each set of experimental conditions was evaluated using the open source software geNorm [50] (http://medgen.ugent.be/~jvdesomp/genorm/). All housekeeping genes received an Expression Stability value (M) of less than 1.5 and were thereby considered adequate reference genes for a particular experiment [50]. All *psbA* and *psaA* values were normalized to the geometric mean of three reference genes (SAM, Actin and PCNA) and expressed relative to untreated (control) samples taken at the same time as the treatment. As previously mentioned, the β-Actin and PCNA primers failed to amplify *Symbiodinium* A20 and D1, respectively. Therefore, these *Symbiodinium* types were only normalized against two of the three housekeeping genes.

### Chlorophyll *a* Fluorescence

Photosynthetic activity during each experiment was assessed by active chlorophyll *a* fluorescence, using a fluorescence induction and relaxation system (FIRe fluorometer, Satlantic), using either the internal cuvette or with a fiber optic probe (for the cultures and corals respectively). Samples were dark-acclimated for 10–20 min, prior to the FIRe measurement protocol, which consisted of a 120 µs saturating single-turnover excitation pulse, followed by 40 weak modulated pulses (60 µs separation) to record the relaxation kinetics of PSII. Ten iterations of each fluorescence trace were averaged into a single transient to increase the signal-to-noise ratio. These data were fit to a biophysical model [51] to determine the values of minimum (F_o_) and maximum (F_m_) single turnover fluorescence, as well as the effective absorption cross-section of PSII (σ_PSII_). σ_PSII_ describes a theoretical “size” of the light-absorbing target, inferred through the probability of a photon being absorbed and used for photochemistry [52]. Maximum quantum yield of PSII ((F_m_−F_o_)/F_m_ = F_v_/F_m_) for the cultured *Symbiodinium* was recorded each sampling day (10:00), whereas the fluorescence measurements of each coral fragment were taken approximately one-hour after sunset (21:00), on each day of the experiment.

### Statistical Analysis

All relative changes in gene expression were analyzed using the relative expression software tool, REST 2009 [53] (http://www.qiagen.com/products/rest2009software.aspx). This software employs a statistical randomization technique to test for up or downregulation of a gene, based on a mean expression value normalized against multiple reference genes, with 10,000 iterations. For each *Symbiodinium* culture, differences in F_v_/F_m_ and σ_PSII_ from the untreated controls (Day 0) were tested using a one-way analysis of variance (ANOVA) in SPSS version 19.0. Data were tested for assumptions of normality (Sharpiro-Wilk) and homogeneity of variance (Levene's test), and when applicable, a Tukey's multiple comparison test analyzed the intraspecific differences of each fluorescence metric. For the *in hospite Symbiodinium* measurements, sample variance was not homogeneous at all time points (Levene's test; p<0.05), and therefore, differences in F_v_/F_m_ and σ_PSII_ between treatment and control corals for a particular *Symbiodinium* type were determined using the nonparametric Mann-Whitney U test.

## Results and Discussion

**Figure 1:**
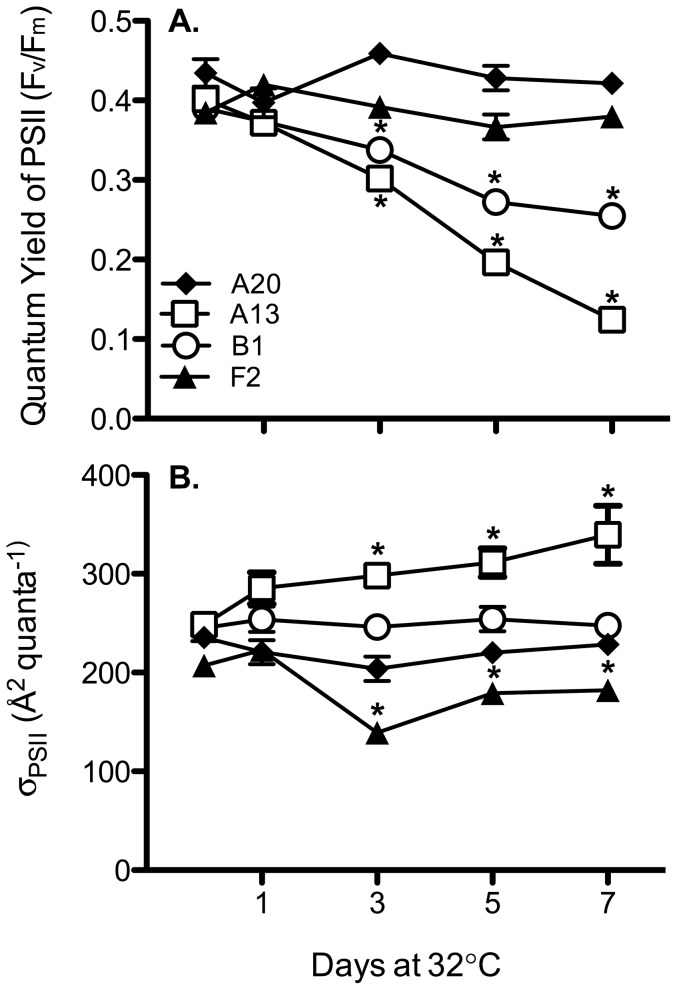
Chlorophyll *a* fluorescence measurements of cultured *Symbiodinium* during the thermal stress treatment. (a.) Maximum quantum yield of PSII (F_v_/F_m_) and (b.) the effective absorption cross-section of PSII (**σ**_PSII_) across four *Symbiodinium* phylotypes, prior to (Day 0) and during (Day 1–7) thermal stress (32**°**C). Asterisks (*) represent statistically significant values that differed from the untreated controls (One-way ANOVA; p<0.05). For each point, n = 4 ±SD.

**Figure 2:**
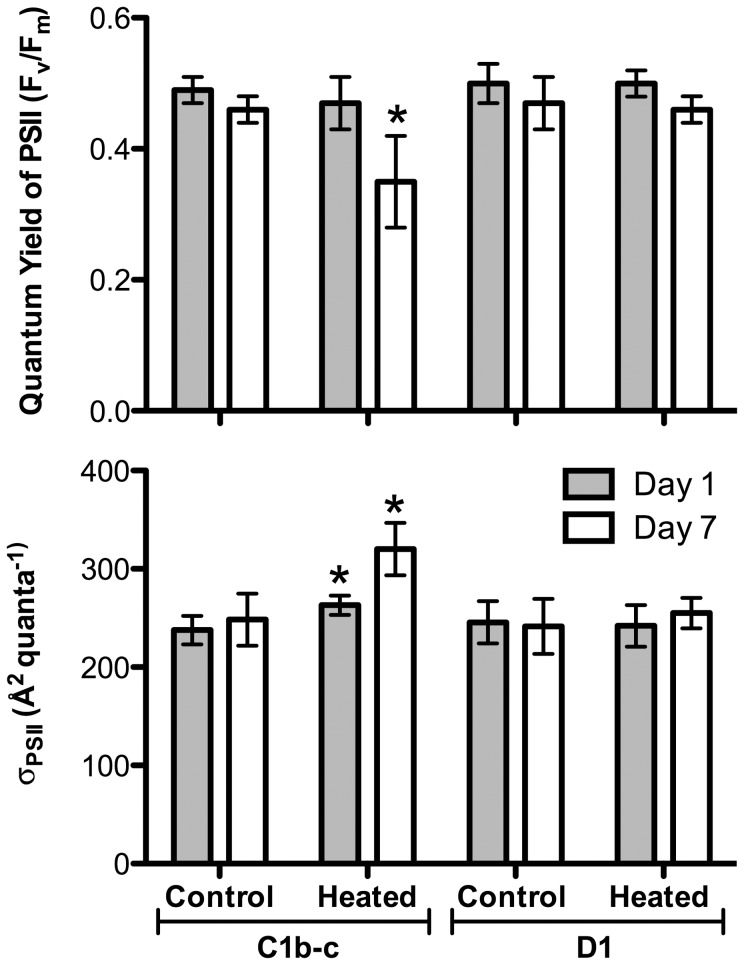
Maximum quantum yield of PSII (F_v_/F_m_) and the effective absorption cross-section of PSII (σ_PSII_) during the simulated bleaching event. (a.) Maximum quantum yield of PSII (F_v_/F_m_) and (b.) the effective absorption cross-section of PSII (**σ**_PSII_) for *Symbiodinium* C1b-c and D1 after Day 1 and 7 of thermal stress (32**°**C). Asterisks (*) represent statistically significant differences between controls and treatments for each *Symbiodinium* type at a time point (Mann-Whitney U test; p**<**0.05). For each point, n = 3–6 **±**SD.

The four cultured *Symbiodinium* isolates differed in their chlorophyll *a* fluorescence in response to elevated temperatures (32°C) (Figure 1). The quantum yield of PSII (F_v_/F_m_) provides insight into the efficiency at which PSII reaction centers are operating within the functional chloroplasts. Exposure to 32°C induced a significant reduction in F_v_/F_m_ for both thermally sensitive culture strains (A13 and B1) beginning on day 3 and continuing to decline with each subsequent sampling point (Figure 1a; p<0.05, ANOVA); whereas, this metric remained relatively stable over the course of the experiment in the thermally tolerant isolates (A20 and F2). Similarly, the *in hospite* chlorophyll *a* fluorescence measurements of *Pocillopora* fragments revealed significant differences between the photochemical competency of *Symbiodinium* C1b-c and D1 exposed to thermal stress (Figure 2). F_v_/F_m_ declined significantly in heated corals associated with *Symbiodinium* C1b-c (Figure 2a; p = 0.016, Mann-Whitney U test), whereas colonies with D1 maintained a high level of photochemical efficiency for the duration of the thermal treatment (p>0.05). Similar to thermal treatments tested by Robison and Warner (2006) with several of these same cultured isolates (i.e. reduced F_v_/F_m_ in B1 and A13 by 29% and 44%, respectively), the significant decline in photochemical efficiency observed in the thermally sensitive types (i.e. A13, B1 and C1b-c) was most likely due to a decline in functional PSII reaction centers and a loss of total D1 protein content [12], [13], [24], [27], [54].

The effective absorption cross-section of PSII (σ_PSII_) also exhibited different patterns amongst the *Symbiodinium* types as the experiment progressed (Figure 1b and 2b). Both A13 and C1b-c displayed significant increases in σ_PSII_ by Day 7 at 32°C (Figure 1b, p<0.01, ANOVA; Figure 2b, p<0.05, Mann-Whitney U test). Under high light stress, Ragni et al. (2010) observed a similar inverse relationship between Fv/Fm and σ_PSII_ in *Symbiodinium* A13. This trend could be explained by a reduction in the content of D1 reaction center proteins that outpaces the loss in antennae pigment binding proteins. While A13 and C1b-c rapidly lose the core of their PSII reaction centers (i.e. D1 protein) under thermal stress [43], its associated light-harvesting pigments may remain well ‘connected’ by continuing to absorb incoming photons and shuttling this energy to other nearby functional reaction centers. This data supports a ‘lake’ model of light-harvesting complex organization in *Symbiodinium* A13 and C1b-c, whereby light harvesting and energy transfer is distributed among several different PSII reaction centers [55], [ but see 36]. Importantly, an increase in σ_PSII_ during thermal stress will lead to greater susceptibility to photoinhibition by elevating the rate of photodamage to the remaining functional PSII [35]. Conversely, the F2 isolate exhibited the opposing pattern whereby F_v_/F_m_ remained stable while σ_PSII_ declined (p<0.05, ANOVA; Figure 1). This trend was likely the result of either a reduction in chlorophyll *a* concentration [43] or a possible change in the xanthophyll content [56]–[58] as the alga begins to acclimate and employ different photoprotective mechanisms under elevated temperature [59]. Overall, the chlorophyll *a* fluorescence data indicates *Symbiodinium* B1, A13, and C1b-c were especially susceptible to thermal stress, undergoing a precipitous breakdown of photochemical efficiency under the elevated temperatures.

**Figure 3:**
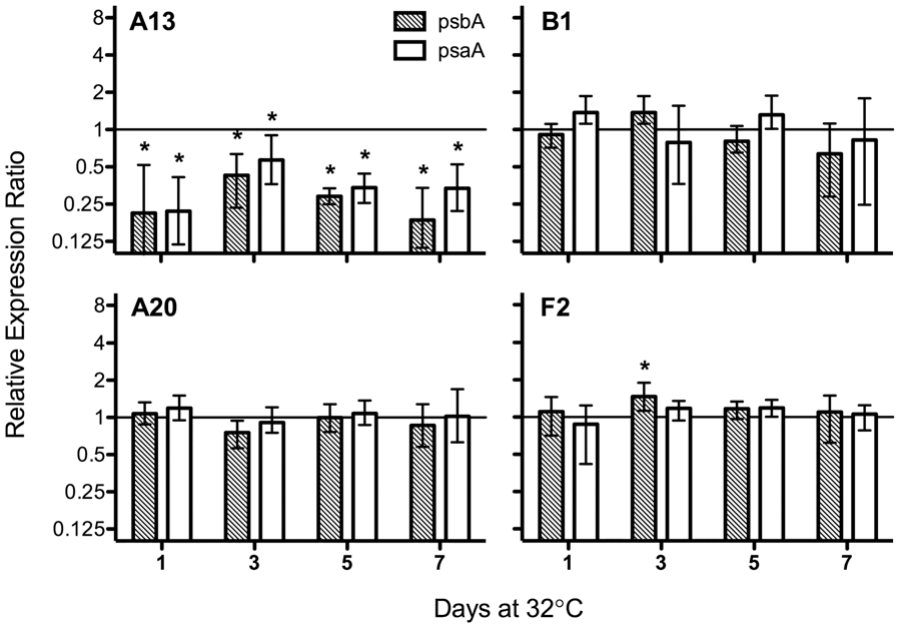
Relative expression of *psbA* and *psaA* across four *Symbiodinium* phylotypes during thermal stress (32°C). Bars represent the mean expression value (logarithmic scale; **±**SE) relative to the untreated controls (28**°**C) for four biological replicates. Asterisks (*) represent values that statistically differed from the untreated controls (REST; p**<**0.05).

**Figure 4:**
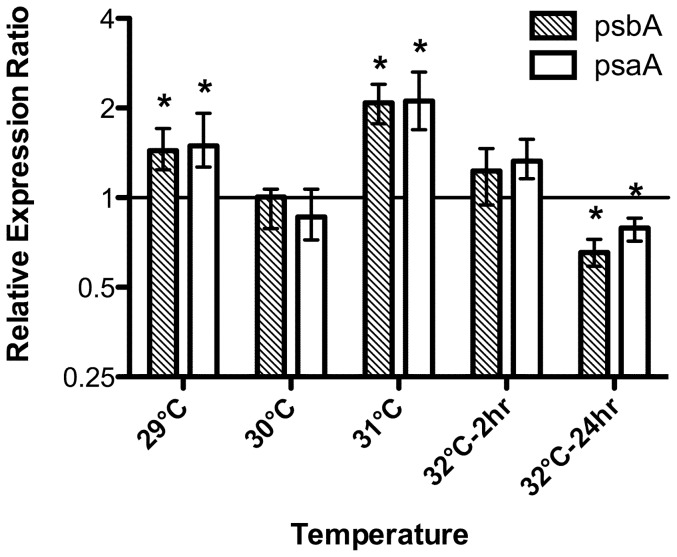
Relative expression of *psbA* and *psaA* for *Symbiodinium* A13 during the temperature ramping procedure (28–32°C). Each bar represents the mean expression value (logarithmic scale; **±**SE) relative to the untreated controls (28**°**C) for four biological replicates. Asterisks (*) represent values that statistically differed from the untreated controls (REST; p**<**0.05).

**Figure 5:**
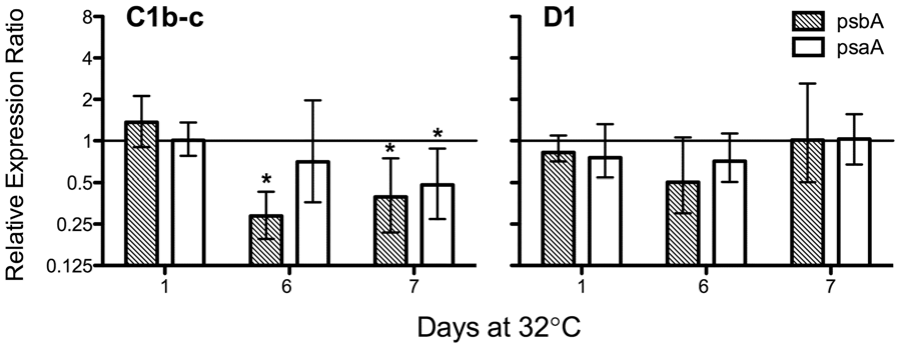
Relative expression of *psbA* and *psaA* for *Symbiodinium* C1b-c and D1 during the simulated bleaching event. Each bar represents the mean expression value (logarithmic scale; **±**SE) relative to the untreated controls (28**°**C) for four biological replicates. Asterisks (*) represent values that statistically differed from the untreated controls sampled the same day (REST; p**<**0.05).

*Symbiodinium* A13 and C1b-c also exhibited a unique trend in their expression of both *psbA* and *psaA*. By 24 h at 32°C, there was a significant decrease (p<0.05, REST) in the transcript abundance of both genes in the A13 isolate that persisted for the remainder of the experiment (Figure 3 and 4). This significant downregulation equates to a persistent loss of transcript abundance ranging from a 2.3–5.4 and a 1.8–4.6-fold decline of *psbA* and *psaA*, respectively (Figure 3). Conversely, transcript levels remained stable for *Symbiodinium* B1, F2, and A20 (Figure 3). Differences in transcript abundance under thermal stress were also observed for intact associations involving *Pocillopora* spp. and its associated symbionts. Here, *Symbiodinium* C1b-c had a considerably lower abundance of *psbA* transcripts on day 6 (3.5-fold decline) and both *psbA* and *psaA* on day 7 at 32°C (*psbA*: 2.6-fold decline; *psaA*: 2.1-fold decline; Figure 5; p<0.05, REST), while plastid transcript abundance in *Symbiodinium* D1 were indistinguishable from the controls (p>0.05, REST). The downregulation of *psbA* and *psaA* in *in hospite Symbiodinium* C1b-c confirms that this stress-induced decline is not solely an artifact from a single unique isolate of cultured *Symbiodinium*. However, the decline in core reaction center transcripts is also not a ubiquitous response that defines all ‘sensitive’ *Symbiodinium* types, as the B1 isolate maintained consistent transcript levels despite an increasing level of photochemical stress (Figure 1 and 3). Considering the differences in photosynthetic properties [60], as well as the variation in the initial site of thermally-induced damage [23], it is likely that the transcriptional response of these genes will differ between some *Symbiodinium* spp., independent of their thermal tolerance.

A closer inspection of *Symbiodinium* A13 during the temperature ramping process revealed that the reduction in the photosynthetic transcripts was only evident after 24 hrs at 32°C (p<0.05, REST; Figure 4). Prior to this time point, *psbA* and *psaA* transcripts were actually upregulated during the temperature ramping process (i.e. 29, 31, and 32°C-2 hrs (*psaA* only); p<0.05, REST). An initial increase of chloroplast transcript abundance, including *psbA* and *psaA*, was also found in green pumpkin cotyledons grown under increasing temperatures until a upper stress-threshold was crossed, which marked a drastic decline in all chloroplast mRNA levels [61]. Elevating photosynthetic transcripts could be an initial acclimation mechanism employed by *Symbiodinium* A13 to adjust to increasing thermal stress. Prior exposure to elevated temperatures can improve the resiliency of both the coral host and the associated *Symbiodinium* community to a subsequent bleaching event [62], [63]. Additionally, as the temperature increases, thermally sensitive *Symbiodinium* lose a significant portion of their functional photosynthetic proteins (e.g. D1 protein and light-harvesting antenna proteins) due to an increased rate of photodamage and/or a reduction in the repair rates of damaged photosynthetic machinery [13], [14], [27], [35], [36]. Perhaps, in addition to a suite of other molecular responses, *Symbiodinium* A13 initially acclimates to increased protein repair rates (or to counteract a reduction in protein repair rates) by upregulating the transcription of *psbA* and *psaA*. Nevertheless, this capacity for acclimation appears limited, as after 24 hrs at 32°C the transcript abundance for both genes was significantly reduced (Figure 3 and 4). A loss of plastid transcripts (including *psbA* and *psaA*) was noted in the green alga, *Euglena gracilis*, and wheat seedlings (*Triticum aestivum*) during heat stress, contributing to an impairment of protein synthesis [64], [65]. Furthermore, the initial downregulation of both *psbA* and *psaA* (Day1 at 32°C; Figure 3) occurred prior to any observed differences in F_v_/F_m_ (Day 3 at 32°C; Figure 1). The sudden decline of both genes suggests that A13 may have reached a critical level of stress prior to the commonly used physiological index of PSII photochemical efficiency. Again, the reduced availability of functional photosynthetic transcripts could greatly hinder the ability of some sensitive *Symbiodinium* spp. to handle periods of thermal stress by introducing another factor limiting the already impaired rates of photosynthetic protein repair [34]–[36].

The relative change of these plastid-encoded genes, in both the cultured and *in hospite* samples, is consistent with other studies measuring the gene expression of several nuclear-encoded transcripts within *Symbiodinium* [46], [66] and other dinoflagellates [67]–[70]. The relatively small differences observed in the mRNA expression of *Symbiodinium* and other dinoflagellates under vastly different conditions has led to the suggestion that most of the changes in protein expression occurs post-transcriptionally [71]–[74]; however, the aforementioned gene expression studies were largely focused on nuclear expression and did not thoroughly evaluate the unique nature of dinoflagellate plastid transcription [39]. Regardless of whether or not *Symbiodinium* possess a strict form of plastid transcriptional control, the reduction of photosynthetic transcripts from two thermally sensitive genotypes, either prior to or simultaneously while other proxies of photoinactivation are noted (e.g. declines in F_v_/F_m_ and D1 protein content), suggests that the availability of chloroplast mRNA could play an important role in the ability of a symbiont to maintain an adequate level of photochemical competency during elevated temperatures. A significant reduction in transcript abundance could act in conjunction with (or even a precursor of) the impaired repair rates of photosynthetic proteins by limiting the substrate availability of the translational machinery. This scenario is especially probable if one considers the exceptionally caustic environment created within a stressed chloroplast [75].

Overall, a stress-induced reduction in chloroplast encoded transcripts could be the result of at least three distinct (yet not necessarily mutually exclusive) processes: (1) an active downregulation of plastid transcription, (2) impairment and/or damage to the transcriptional machinery within the chloroplast, (3) or the degradation of the available functional transcripts. All three processes may act simultaneously and could be initiated by similar underlying factors. For example, under elevated temperatures, thermally sensitive *Symbiodinium* undergo a physiological breakdown and/or damage to several photosynthetic components, such as the D1 protein of PSII, the thylakoid membrane, or inhibition to Rubisco [15], [17], [27]. This impairment of efficient photosynthesis results in a drastic reduction of ATP synthesis, along with a concurrent elevation of reactive oxygen species (ROS) [17], [42], [75]–[77]. Under such “energy-starved” conditions, *Symbiodinium* A13 and C1b-c could actively downregulate transcription of *psbA* and *psaA* due to a decline in the available ATP energy reserves, or in order to conserve the remaining ATP for other vital cellular processes. Furthermore, the concurrent production of excess ROS is extremely destructive to many cellular constituents, including lipids, proteins, and nucleic acids [75]. These conditions create a caustic environment within a stressed chloroplast that could impair the transcriptional machinery or damage the available photosynthetic transcripts. Regardless of the exact underlying mechanism, the decline in these photosynthetic transcripts (especially *psbA*) for *Symbiodinium* A13 or C1b-c could significantly impair their ability to cope with periods of thermal stress. The reduction in plastid-encoded transcripts could limit the substrate availability of the translational machinery and work in conjunction with either lower repair rates [27], [35], [36], [60] or increased damage of photosynthetic proteins [13] to render certain *Symbiodinium* spp. exceptionally susceptible to thermal stress.

## Supporting Information

Figure S1 **Daily sinusoidal light conditions (08:00–22:00) used during the thermal treatment of cultured** ***Symbiodinium*** **.** Filled boxes represent the dark period of each light cycle.
(TIF)
