# Supplementary figures and images for: Transcriptional Response of Two Core Photosystem Genes in Symbiodinium spp. Exposed to Thermal Stress
Source: PLoS One. 2012 Dec 7;7(12):e50439. doi: 10.1371/journal.pone.0050439 (PMC3517614; doi:10.1371/journal.pone.0050439)

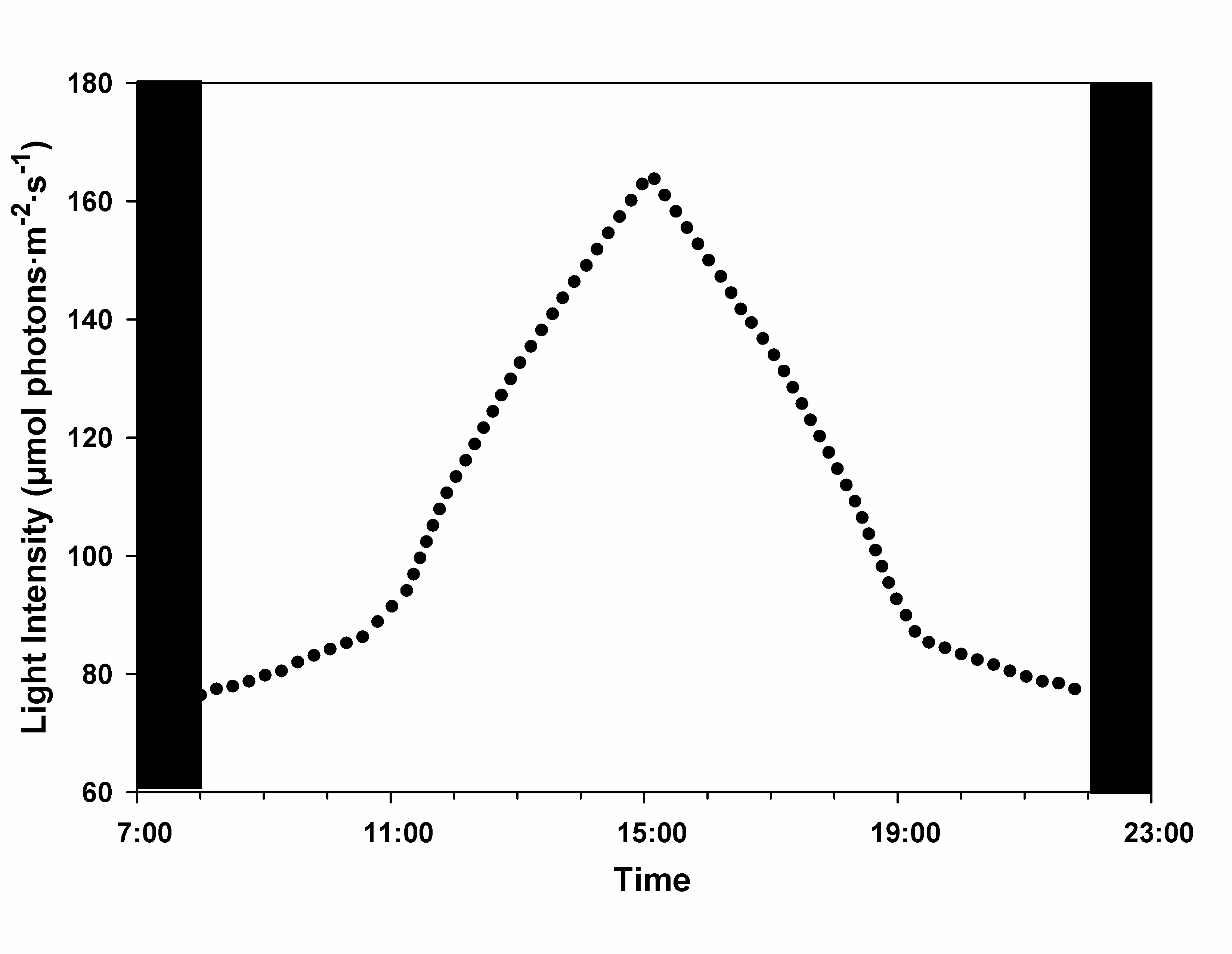

Supplement: Figure S1 — Daily sinusoidal light conditions (08:00–22:00) used during the thermal treatment of cultured Symbiodinium . Filled boxes represent the dark period of each light cycle. (TIF) [file pone.0050439.s001.tif]
